# *TP73-AS1* rs3737589 Polymorphism is Associated With the Clinical Stage of Colorectal Cancer

**DOI:** 10.1155/2023/3931875

**Published:** 2023-02-22

**Authors:** Yichang Gao, Shulong Zhang, Xueren Gao

**Affiliations:** ^1^School of Pharmacy, Nanjing University of Chinese Medicine, Nanjing 210023, China; ^2^Department of General Surgery, Shanghai Xuhui Central Hospital, Zhongshan-Xuhui Hospital, Fudan University, Shanghai 200030, China; ^3^School of Pharmacy, Yancheng Teachers' University, Yancheng 224007, China

## Abstract

**Objective:**

TP73-AS1 can promote the occurrence and development of a variety of tumors, including colorectal cancer (CRC). The current study aimed to investigate the association between a potentially functional genetic polymorphism (rs3737589 T > C) on the *TP73-AS1* gene and the susceptibility and clinical stage of CRC in a Chinese Han population.

**Methods:**

The polymorphic genotyping was performed by the SNaPshot method. The real-time quantitative PCR method and the luciferase assay were used separately to explore genotype-tissue expression and the function of the genetic polymorphism.

**Results:**

A total of 576 CRC patients and 896 healthy controls were included in the current study. The rs3737589 polymorphism was not associated with CRC susceptibility but was associated with the CRC stage (CC vs. TT: OR = 0.25, 95% CI = 0.12 – 0.54, *P*=0.0003; C vs. T: OR = 0.69, 95% CI = 0.53–0.89, *P*=0.006; and CC vs. (TC + TT): OR = 0.26, 95% CI = 0.12–0.56, *P*=0.0004). CRC patients carrying the rs3737589 CC genotype or C allele were less likely to have stage III/IV tumors than those carrying the rs3737589 TT genotype or T allele. The expression of TP73-AS1 was lower in CRC tissues with the rs3737589 CC genotype compared to those with the TT genotype. Bioinformatics analysis and the luciferase assay revealed that the C allele could promote the binding of miR-3166 and miR-4771 to TP73-AS1.

**Conclusion:**

The *TP73-AS1* gene rs3737589 polymorphism affecting miRNAs binding is associated with the CRC stage and may serve as a biomarker for predicting CRC progression.

## 1. Introduction

Colorectal cancer (CRC) is the third most common malignant tumor and the main cause of cancer death in the world [1]. The occurrence and development of CRC is a complicated and multidimensional process, and epidemiological studies have found multiple risk factors associated with the occurrence of CRC, including various unhealthy diets and lifestyles [2]. In addition, previous genomics studies have revealed that single nucleotide polymorphisms (SNPs) in several CRC-related genes are linked to the risk and progression of CRC [3–8]. For instance, the *interleukins-17A* (*IL-17A*) rs2275913 G > A polymorphism was associated with the occurrence and severity of CRC in the Bulgarian population [6]. Three SNPs (rs2094258 C > T, rs751402 C > T, and rs873601 G > A) on the xeroderma pigmentosum group G (XPG) gene were found to associate with CRC susceptibility in a Southern Chinese population [8].

Long noncoding RNA (lncRNA) is a class of RNA that is more than 200 nucleotides in length and has no obvious protein-coding capacity. LncRNA can be involved in the occurrence and development of many diseases, including tumors [9–12]. For instance, lncRNA TP73-AS1 transcribed from chromosome 1p36 has been identified as a novel oncogenic molecule in many tumors, such as ovarian cancer, cervical cancer, hepatoma, retinoblastoma, breast cancer, gastric cancer, and CRC [13–19]. Given the important role of TP73-AS1 in tumors, several studies have recently begun to investigate the relationship between SNPs on the *TP73-AS1* gene and tumor susceptibility. Fan et al. found that the *TP73-AS1* gene rs9800 polymorphism was significantly related to CRC risk [20]. Chen et al. found that the *TP73-AS1* gene rs3737589 polymorphism might be associated with the risk of gastric cancer [21]. In addition, the rs3737589 polymorphism was a potential biomarker to predict the prognosis of patients with gastric cancer [21]. Considering that the role of the rs3737589 polymorphism in CRC is still unknown, we analyzed the association of the *TP73-AS1* gene rs3737589 polymorphism with the susceptibility and clinical stage of CRC in the current study.

## 2. Materials and Methods

### 2.1. Study Population

Peripheral blood samples from 576 CRC patients and 896 healthy controls were recruited from Shanghai Xuhui District Central Hospital. Patients were pathologically diagnosed with CRC. Healthy controls recruited from medical examinations had no history of cancer, no intestinal disease, and no systemic disease. In addition, CRC and normal paracancerous tissues were obtained from 50 CRC surgery patients who had not received radiochemotherapy before surgery. All individuals were genetically unrelated to ethnic Han Chinese.

### 2.2. Genotyping

Genomic DNA was extracted from all peripheral blood samples using the TIANamp Genomic DNA Kit according to the manufacturer's instructions. The genotyping of the rs3737589 polymorphism was performed by the SNaPshot method. To verify the accuracy of the genotyping results, 10% of the DNA samples were randomly selected for direct sequencing. The concordance of sequencing results was 100%.

### 2.3. Real-Time Quantitative PCR and Bioinformatics Analysis

Total RNA was isolated from CRC and normal paracancerous tissues using the RNAsimple total RNA kit (Tiangen) according to the manufacturer's instructions. ReverTra Ace qPCR RT Master Mix Kit (Toyobo) was used to synthesize cDNA. FastStart Universal SYBR Green Master (Roche) was used to conduct real-time quantitative PCR. The expression of TP73-AS1 was normalized to the internal control GAPDH. The 2^−ΔΔCt^ method was used to calculate gene relative expression. The specific primer sequences are presented in Table 1. In addition, the GTEx database (https://www.gtexportal.org/home/) was also used to analyze the expression of TP73-AS1 in colon tissue of different rs3737589 genotypes [22]. The lncRNASNP v3 database (https://gong_lab.hzau.edu.cn/lncRNASNP3/) was used to analyze the effect of the rs3737589 polymorphism on miRNA binding [23]. To assess the influence of the rs3737589 polymorphism on the secondary structure of TP73-AS1, the RNAfold web server (https://rna.tbi.univie.ac.at/cgi-bin/RNAWeb%20Suite/RNAfold.cgi) was used to draw a plain structure and mountain plot of TP73-AS1 (NR_033708.1) based on the minimum free energy [24].

### 2.4. Luciferase Assay

A 200 bp sequence carrying rs3737589 C or T allele was inserted into the psiCHECK2 vector and then cotransfected with miR-3166, miR-4771, or miRNA-NC, respectively, into 293T cells using Liposome 2000. Transfected cells were collected 48 hours after transfection and their luciferase activity was evaluated using a dual-luciferase reporter assay kit and a luminometer. Each test was performed in triplicate and at least three times.

### 2.5. Statistical Analysis

Hardy–Weinberg equilibrium (HWE) for the control group was tested by a goodness-of-fit *χ*^2^ test. The association of the rs3737589 polymorphism with CRC susceptibility was evaluated using adjusted odds ratios (ORs) with their 95% confidence intervals (CIs). The Student's *t*-test was used to check the difference in age variable between CRC patients and healthy controls, and compare the relative luciferase activity of different alleles. *χ*^2^ test was used to assess the difference in gender variables between CRC patients and healthy controls. In the real-time quantitative PCR experiment, TP73-AS1 expression among different genotypes was compared using one-way ANOVA. All statistical analyses were performed by SAS 9.4 (SAS Institute, Cary, USA). *P* < 0.05 was defined as the level of significance.

## 3. Results

### 3.1. Association between the rs3737589 Polymorphism and the Susceptibility and Clinical Stage of CRC

The basic characteristics of CRC patients and healthy controls were shown in Table 2. The age and gender distributions in the case and control groups did not differ statistically. The genotype frequency distribution of the control group was consistent with HWE (Table 3). There was no link found between the rs3737589 polymorphism and CRC susceptibility (Table 3). However, the analysis based on the clinical stage showed that the rs3737589 polymorphism was associated with the CRC stage (Table 4). CRC patients carrying the rs3737589 CC genotype or C allele were less likely to have stage III/IV tumors than those carrying the TT genotype or T allele (CC vs. TT: OR = 0.25, 95% CI = 0.12–0.54, *P*=0.0003; C vs. T: OR = 0.69, 95% CI = 0.53–0.89, *P*=0.006). In addition, this significant association was also present under the recessive model (CC vs. (TC + TT): OR = 0.26, 95% CI = 0.12–0.56, *P*=0.0004).

### 3.2. Genotype-Tissue Expression

**Figure 1:**
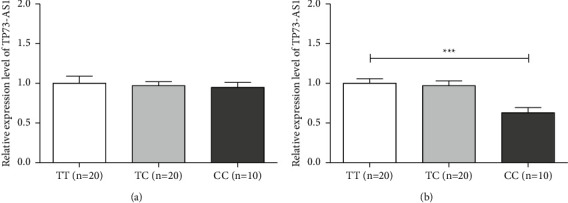
Genotype-tissue expression analysis of the rs3737589 polymorphism in colorectal tissues: (a) normal paracancerous tissues; (b) CRC tissues.

**Figure 2:**
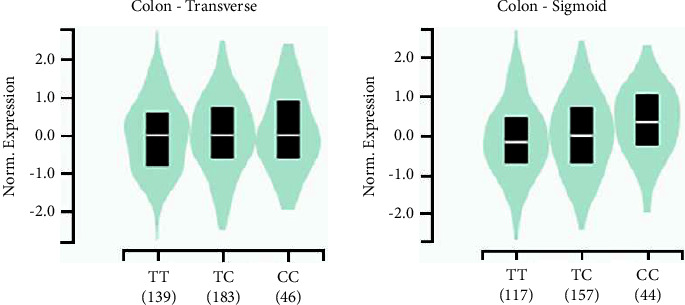
Genotype-tissue expression analysis of the rs3737589 polymorphism in colon tissues from the GTEx database.

The rs3737589 polymorphism was not associated with TP73-AS1 expression in normal paracancerous tissues but was significantly associated with TP73-AS1 expression in CRC tissues (Figure 1). TP73-AS1 expression was significantly lower in CRC tissues with the CC genotype compared to those of the TT genotype. In addition, TP73-AS1 expression was significantly higher in colon tissues with the rs3737589 CC genotype (Figure 2).

### 3.3. Bioinformatics Analysis and the Luciferase Assay

**Figure 3:**
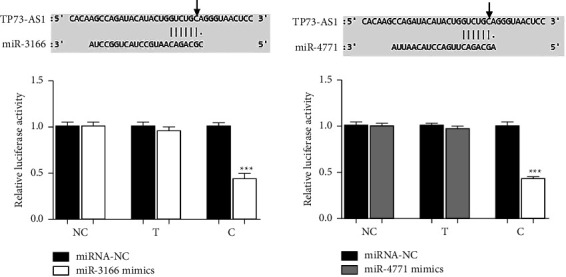
The effect of the rs3737589 polymorphism on miRNA binding.

**Figure 4:**
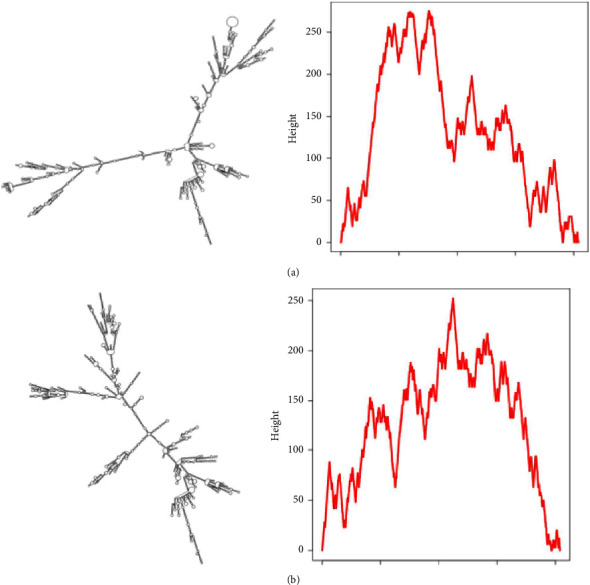
The influence of the rs3737589 polymorphism on the secondary structure of TP73-AS1: (a) TP73-AS1 with the rs3737589 A allele; (b) TP73-AS1 with the rs3737589 G allele.

The rs3737589 C allele contributed to the binding of miR-3166 and miR-4771 to TP73-AS1 (Figure 3). In addition, the rs3737589 polymorphism could affect the secondary structure of TP73-AS1 (Figure 4).

## 4. Discussion

Cai et al. found that TP73-AS1 could sponge miR-194 to promote CRC cell proliferation, migration, and invasion by up-regulating TGF*α* [19]. Jia et al. observed that TP73-AS1 could enhance CRC proliferation by functioning as a ceRNA for miR-103, which controlled PTEN expression [25]. Furthermore, Li et al. found that TGF-*β*1 could be activated by TP73-AS1 to promote CRC cell migration and invasion [26]. These previous findings suggested that TP73-AS1 could promote the progression of CRC. Since SNPs on cancer-associated lncRNA genes might be involved in the occurrence and development of cancers, we evaluated the association of a potentially functional polymorphism (rs3737589) on the *TP73-AS1* gene with CRC susceptibility and clinical stage and found no significant association between this genetic polymorphism and CRC susceptibility. However, the rs3737589 polymorphism was associated with the CRC stage. CRC patients carrying the rs3737589 CC genotype or C allele were less likely to have stage III/IV tumors than those carrying the TT genotype or T allele. Further analysis showed that TP73-AS1 expression was significantly lower in CRC tissues with the rs3737589 CC genotype compared to those of the TT genotype. The C allele for this genetic polymorphism located on the TP73-AS1 transcript could promote the binding of miR-3166 and miR-4771. Therefore, we speculated that the rs3737589 polymorphism might influence the regulation of TP73-AS1 expression by miR-3166 and miR-4771 and thus associated CRC progression. In addition, the rs3737589 polymorphism might also affect TP73-AS1 stability by altering the structure of TP73-AS1 and thus associating CRC progression.

Although the current study has made some interesting findings, there are still some issues that need improvement. GTEx data showed that the rs3737589 polymorphism was associated with TP73-AS1 expression in colon tissues, which was not confirmed by the current study due to the limited sample size. Since information on the lifestyle and diet of the studied individuals and clinical data were not fully collected, the study did not adjust for other confounding factors such as smoking, alcohol consumption, and red meat intake. In addition, the current study did not elucidate the exact function of the rs3737589 polymorphism in CRC. Thus, more studies are needed to confirm our findings.

In conclusion, our study confirmed that the *TP73-AS1* rs3737589 polymorphism was not associated with CRC susceptibility in the Chinese Han population, but was associated with the CRC stage. This polymorphism may serve as a biomarker for predicting CRC progression.

## Figures and Tables

**Table 1:** The specific primer sequences for real-time quantitative PCR.

Genes	Primer sequences
TP73-AS1	Forward: 5′-ACTCCGGACACTGTGTTTTCTC-3′
	Reverse: 5′-GCATCTTTTAAGGCGGCCATATC-3′

GAPDH	Forward: 5′-GTCTCCTCTGACTTCAACA-3′
	Reverse: 5′-TGAGGGTCTCTCTCTTCCT-3′

**Table 2:** Basic characteristics of CRC patients and healthy controls.

Variables	CRC patients (*N* = 576)	Healthy controls (*N* = 896)	*P* value
Age, mean ± SD	59.4 ± 7.3	59.2 ± 6.4	0.54
Gender			
Male	351 (60.9%)	516 (57.6%)	0.20
Female	225 (39.1%)	380 (42.4%)	
Clinical stage			
I + II	300 (52.1%)		
III + IV	276 (47.9%)		

**Table 3:** Association between *TP73-AS1* gene rs3737589 polymorphism and CRC susceptibility.

Genotype or allele	CRC patients (*N* = 576)	Healthy controls (*N* = 896)	^*a*^OR (95% CI)	^*a*^ *P* value
Genotype				
TT	318 (55.2%)	508 (56.7%)	Reference	
TC	213 (37.0%)	333 (37.2%)	1.03 (0.82–1.28)	0.85
CC	45 (7.8%)	55 (6.1%)	1.33 (0.87–2.03)	0.22
Ptrend				0.36
PHWE				0.97
TT	318 (55.2%)	508 (56.7%)	Reference	0.59
TC + CC	258 (44.8%)	388 (43.3%)	1.07 (0.86–1.32)	
TT + TC	531 (92.2%)	841 (93.9%)	Reference	0.22
CC	45 (7.8%)	55 (6.1%)	1.32 (0.88–1.99)	
Allele				
T	849 (73.7%)	1349 (75.3%)	Reference	0.33
C	303 (26.3%)	443 (24.7%)	1.09 (0.92–1.29)	

^*a*^Adjusted for age and gender.

**Table 4:** The association between *TP73-AS1* gene rs3737589 polymorphism and clinical stage of CRC.

Genotype or allele	III + IV (*n* = 276)	I + II (*n* = 300)	Comparison	^*a*^OR (95% CI)	^*a*^ *P* value
Genotype					
TT	161 (58.3%)	157 (52.3%)	TC vs. TT	0.98 (0.69–1.38)	0.96
TC	106 (38.4%)	107 (35.7%)	CC vs. TT	0.25 (0.12–0.54)	0.0003
CC	9 (3.3%)	36 (12.0%)	C vs. T	0.69 (0.53–0.89)	0.006
Allele					
T	428 (77.5%)	421 (70.2%)	(TC + CC) vs. TT	0.79 (0.57–1.10)	0.19
C	124 (22.5%)	179 (29.8%)	CC vs. (TC + TT)	0.26 (0.12–0.56)	0.0004

^*a*^Adjusted by age and gender.

## Data Availability

The data used to support this study are available from the corresponding author upon request.
